# Clinical Characteristics and Outcomes of Surgical Treatment of Solitary Osteochondromas in Children: A 11-Year Retrospective Study

**DOI:** 10.3390/medsci14040444

**Published:** 2026-07-27

**Authors:** Zenon Pogorelić, Mladen Banović, Ivan Lovrinčević, Sandra Zekić Tomaš, Klaudio Pjer Milunović

**Affiliations:** 1 Department of Pediatric Surgery, University Hospital of Split, Spinčićeva 1, 21000 Split, Croatia; 2 Department of Surgery, School of Medicine, University of Split, Šoltanska 2A, 21000 Split, Croatia; 3 Department of Pathology, Forensic Medicine and Cytology, University Hospital of Split, Spinčićeva 1, 21000 Split, Croatia; 4 Department of Pathology, School of Medicine, University of Split, Šoltanska 2A, 21000 Split, Croatia

**Keywords:** osteochondroma, exostosis, children, bone tumor, surgical excision, postoperative outcomes, recurrence

## Abstract

Background: Osteochondroma is the most common benign bone tumor in childhood. Although most lesions are asymptomatic, surgical treatment is indicated in patients with pain, mechanical symptoms, cosmetic concerns, restricted range of motion, or neurovascular compression. This study evaluated the clinical characteristics and surgical outcomes of osteochondromas in children and adolescents treated at a single tertiary pediatric surgery center. Methods: This retrospective study included 75 pediatric patients who underwent surgical excision of osteochondroma between January 2015 and January 2026. Demographic, clinical, radiological, operative, histopathological, and follow-up data were analyzed. Lesion-related variables were assessed at the level of individual exostoses. The primary outcome was surgical treatment outcome, while secondary outcomes included lesion localization, symptoms, hospital stay, postoperative complications, recurrence, and associations between clinical and morphological variables. Results: A total of 75 patients with 76 solitary osteochondromas were included. The median age was 13 years, and 64.0% of patients were male. Most lesions were located distally (73.7%), with the femur being the most common site (48.7%), followed by the tibia (22.4%). Pain was the most frequent symptom, present in 63.2% of exostoses, followed by cosmetic concern in 32.9%. Standard radiography was used in nearly all cases (98.7%). Radiographic measurements significantly underestimated lesion size compared with intraoperative measurements, with median diameters of 3.0 cm and 4.0 cm, respectively (*p* < 0.001). A strong positive correlation was observed between radiographic and intraoperative lesion diameter (ρ = 0.77; *p* < 0.001), while symptom duration showed a weak but significant correlation with lesion diameter (ρ = 0.28; *p* = 0.013). No significant association was found between lesion diameter and symptomatic presentation, and logistic regression did not identify age, sex, lesion location, or diameter as significant predictors of symptoms. Postoperative outcomes were favorable: 94.6% of patients had no complications, wound infection and hematoma occurred in 2.7% each, and no recurrences or reoperations were recorded during a median follow-up of 63 months. Histopathological examination confirmed osteochondroma in all cases. Conclusions: Surgical excision of symptomatic pediatric osteochondromas was associated with favorable postoperative outcomes, a low complication rate, and no observed recurrence during follow-up. Standard radiography correlates well with intraoperative findings but may underestimate true lesion size, likely due to limited visualization of the cartilaginous cap. Symptom development appears multifactorial and cannot be reliably predicted by lesion size or location alone.

## 1. Introduction

Osteochondroma represents the most common benign bone tumor in childhood, accounting for 35–50% of all benign bone tumors [1,2]. It arises from the metaphyseal regions of long bones as a consequence of disrupted enchondral ossification, resulting in a bony outgrowth covered by a cartilaginous cap [1,2]. Osteochondromas occur either as solitary lesions or, less commonly, as part of hereditary multiple osteochondromas (HMO), an autosomal dominant disorder associated with mutations in the EXT1 and EXT2 genes [3,4,5,6].

Osteochondromas are typically diagnosed during childhood and adolescence, with peak incidence between 10 and 14 years of age, and occur more frequently in males [1,2]. The distal femur, proximal tibia, and proximal humerus are the most common anatomical locations [2]. Although many lesions remain asymptomatic and are discovered incidentally, others may cause pain, mechanical symptoms, restriction of movement, cosmetic deformity, or neurovascular compression depending on their size and anatomical relationship to surrounding structures [2,7,8]. These manifestations represent the principal indications for surgical treatment.

Standard radiography remains the primary imaging modality because it demonstrates continuity of the cortex and medullary canal between the lesion and the parent bone [9]. CT and particularly MRI are reserved for selected cases requiring assessment of the cartilage cap, neurovascular compression, or suspected complications [2,9]. Although malignant transformation is uncommon, particularly in solitary lesions, evaluation of cartilage cap thickness remains important when radiological or clinical findings raise suspicion for malignancy [4,9,10,11].

Management depends primarily on clinical presentation. Asymptomatic osteochondromas are generally managed conservatively with clinical and radiological follow-up, whereas symptomatic lesions are treated surgically [7,12]. Complete surgical excision, including removal of the cartilage cap and perichondrium, has been associated with low recurrence rates and favorable postoperative outcomes [7,13].

Despite generally favorable outcomes, evidence regarding predictors of symptom development, the relationship between lesion morphology and clinical presentation, and long-term surgical outcomes in children remains limited [2,7]. Therefore, the aim of this study was to evaluate the clinical characteristics and surgical outcomes of children with solitary osteochondromas treated at a tertiary pediatric center and to explore associations between lesion morphology, symptoms, and operative findings.

## 2. Methods

### 2.1. Patients

**Figure 1:**
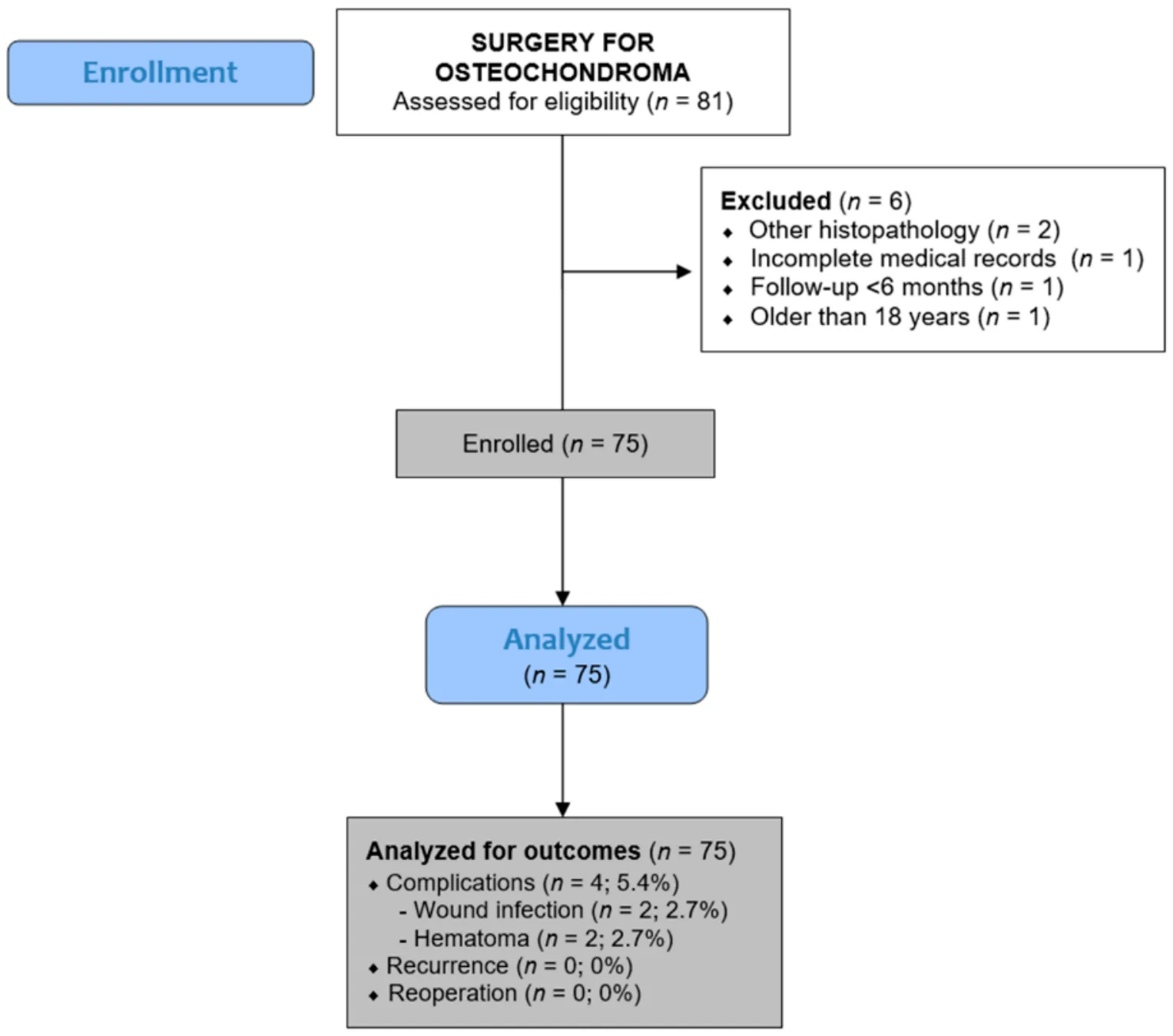
Flow-chart of the study.

The medical records of 75 pediatric patients who underwent surgery for osteochondroma at the Department of Pediatric Surgery, University Hospital of Split between 1 January 2015 and 1 January 2026 were evaluated. All patients aged 0–17 years who underwent surgical excision of osteochondroma during the study period were eligible for inclusion, regardless of lesion location (extremities, trunk, or pelvis) and irrespective of whether the lesion was solitary or multiple (hereditary multiple osteochondromas). Exclusion criteria included patients older than 18 years, patients with incomplete medical records, patients with a different histopathological diagnosis, and patients with a follow-up period of less than 6 months after surgery were excluded. The indication for surgical treatment was established by the attending pediatric surgeon responsible for the patient’s care in consultation with the patient’s parents or legal guardians. Surgical indications followed the institutional practice and included pain, mechanical symptoms, cosmetic concerns, restricted range of motion, and neurovascular compression. The flow chart of the study is shown in Figure 1.

### 2.2. Ethical Aspects

This study was conducted in accordance with the Declaration of Helsinki of the World Medical Association and its subsequent amendments. Approval was obtained from the Institutional Review Board of the University Hospital of Split (approval number: 520-03/25-01/256; date of approval: 28 November 2025). The requirement for informed consent for participation in this retrospective study was waived by the Institutional Ethics Committee.

### 2.3. Study Outcomes

The primary outcome of this retrospective study was to evaluate the outcomes of surgical treatment in children with osteochondroma. Secondary outcomes included the analysis of demographic and clinical characteristics, localization of osteochondromas, length of hospital stay, and the incidence of postoperative complications. Furthermore, the relationship between clinical and morphological characteristics of exostoses was assessed, including the comparison between radiographic and intraoperative measurements, as well as the association between symptom duration and lesion size. In addition, potential predictors of symptom occurrence were evaluated using logistic regression analysis.

### 2.4. Study Design

Data were collected through a retrospective review of hospital records and the electronic medical database (Hospital Information System of the University Hospital of Split). The following variables were extracted: patient demographics (age, sex, height, body weight, and body mass index), comorbidities, and American Society of Anesthesiologists (ASA) classification. Clinical and lesion-related characteristics included the size and localization of osteochondromas, radiographic and intraoperative diameter of the lesions, presenting symptoms, duration of symptoms, and type of lesion (solitary or multiple). Analyses of lesion-related variables were performed at the level of individual exostoses. Accordingly, patient-level analyses included 75 patients, whereas lesion-level analyses included 76 exostoses because one patient underwent excision of two separate lesions. Treatment-related variables included the duration of surgery and length of hospital stay, while outcome measures comprised postoperative complications, recurrence rate, duration of follow-up, and histopathological findings. Imaging modalities used for diagnosis, such as standard radiography, computed tomography (CT), and magnetic resonance imaging (MRI) were also recorded. Radiographic measurements were performed retrospectively and independently by two pediatric surgeons using the original preoperative plain radiographs available in the institutional Picture Archiving and Communication System (PACS). The maximum longitudinal diameter of each osteochondroma was measured on the projection that best demonstrated the lesion. All data were analyzed anonymously, without patient identifiers. For standardization purposes, postoperative complications were classified according to the Clavien–Dindo classification [14], and recurrence was defined as the reappearance of a lesion at the same anatomical site during the follow-up period. All collected data were stored in a secure electronic database accessible only to the investigators involved in the study.

### 2.5. Surgical Procedure

**Figure 2:**
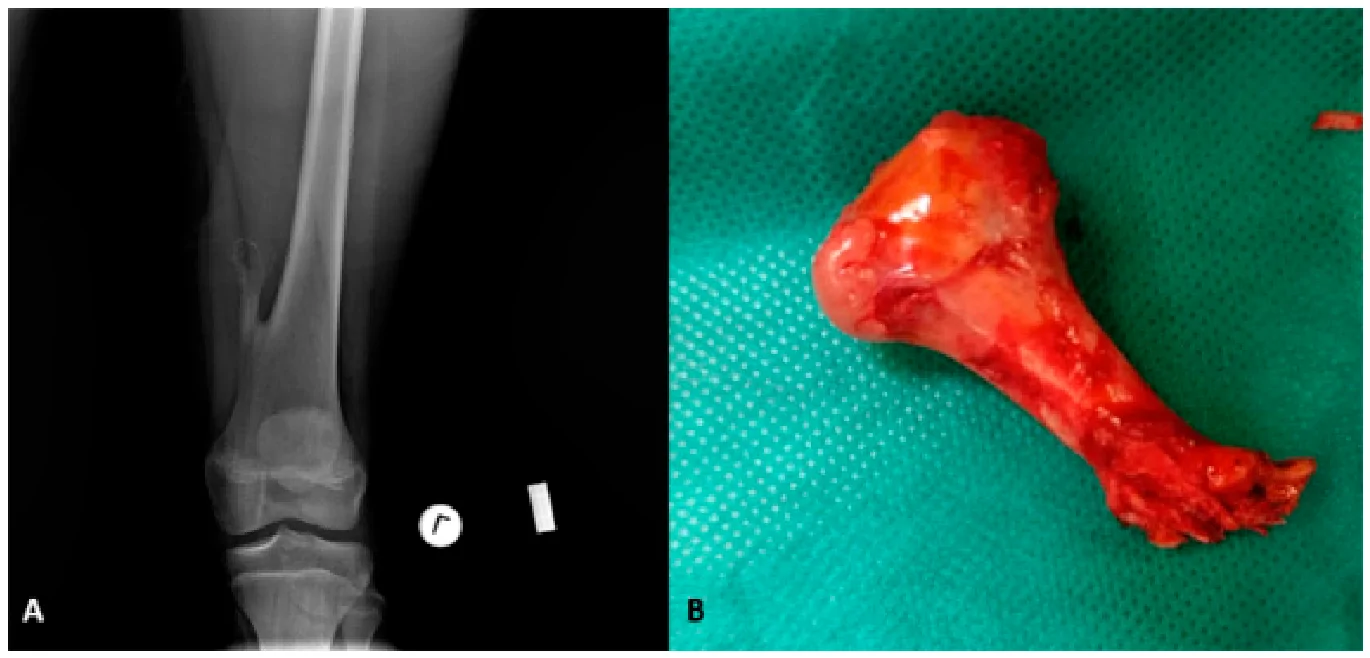
Osteochondroma of the distal femur in a 12-year-old girl. (**A**) Preoperative radiograph demonstrating a well-defined exophytic bone lesion arising from the distal femur; (**B**) Intraoperative view showing the lesion after surgical exposure and complete excision.

**Figure 3:**
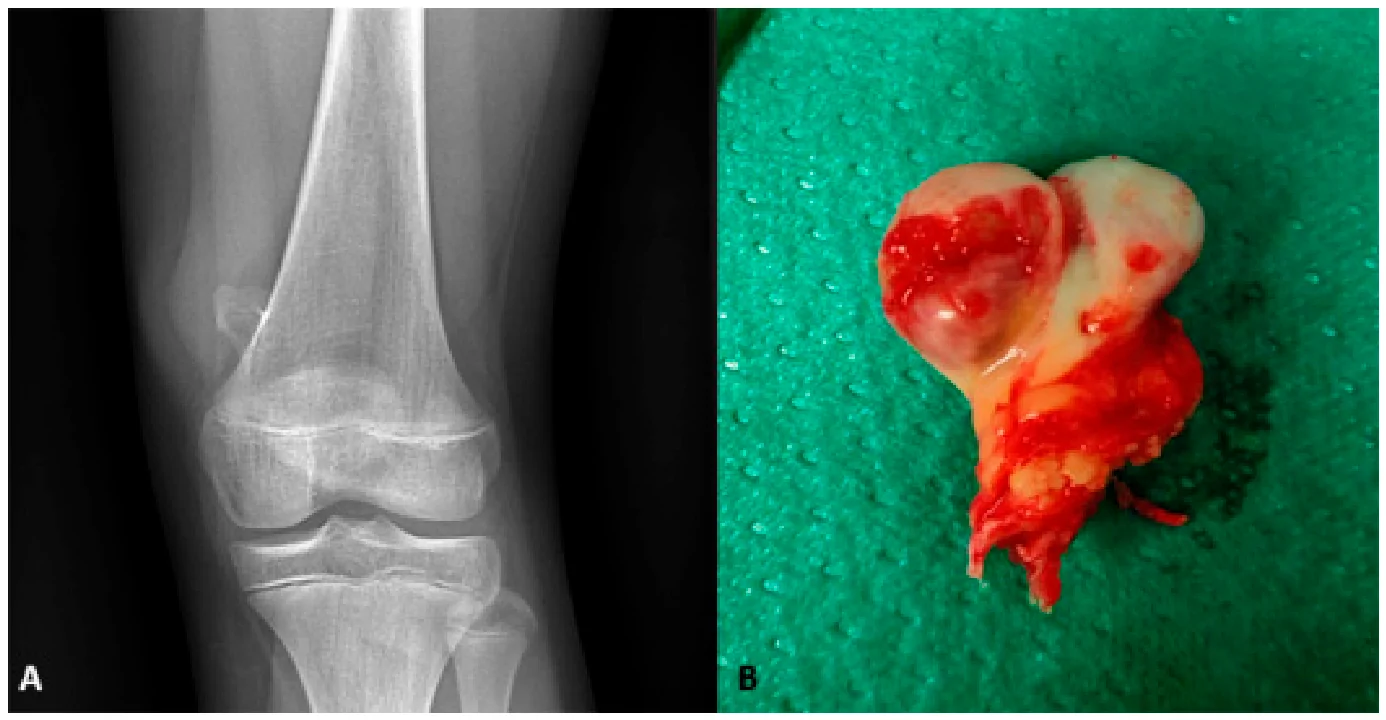
Osteochondroma of the distal femur in a 14-year-old male adolescent. (**A**) Preoperative radiograph demonstrating a well-defined exophytic bone lesion arising from the distal femur; (**B**) Intraoperative view showing the lesion after surgical exposure and complete excision.

**Figure 4:**
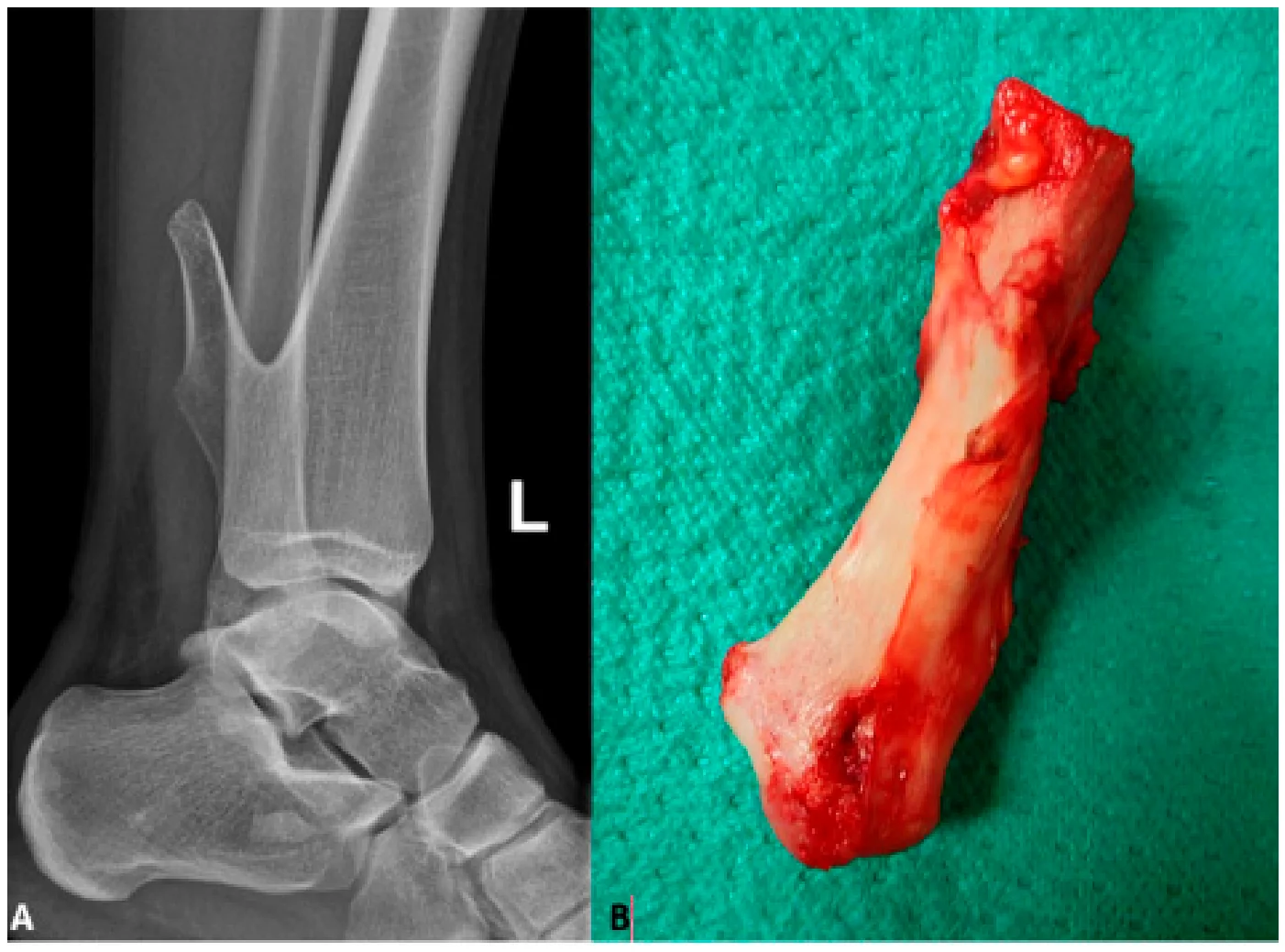
Osteochondroma of the distal tibia in a 16-year-old male adolescent. (**A**) Preoperative radiograph demonstrating a large well-defined exophytic lesion arising from the distal tibia; (**B**) Intraoperative view showing the lesion following surgical exposure and complete excision.

All procedures were performed under general anesthesia by one of six board-certified pediatric surgeons using the same standardized institutional surgical technique. A longitudinal skin incision was made over the lesion, followed by careful dissection through the soft tissues to expose the osteochondroma. Particular attention was paid to the protection of adjacent neurovascular structures. The lesion was excised en bloc at its base, including the cartilage cap and a thin margin of surrounding cortical bone, in order to minimize the risk of recurrence. Representative examples of preoperative radiographic imaging, intraoperative findings, and corresponding histopathological specimens are presented in Figure 2, Figure 3 and Figure 4. All excised specimens were routinely sent for histopathological analysis. The wound was irrigated and closed in layers. Postoperative care followed a standardized protocol. Prophylactic antibiotics were not routinely administered. Patients were discharged once adequate pain control was achieved, wound status was satisfactory, and mobilization was possible without significant difficulty.

### 2.6. Histopathological Examination

All excised specimens were submitted for routine histopathological examination. After fixation in formalin, the specimens were processed according to standard institutional protocols. Representative sections were examined by an experienced pathologist to confirm the diagnosis of osteochondroma and to exclude other bone lesions or features suspicious for malignant transformation. The maximum diameter of the excised specimen was routinely measured and recorded by an experienced pathologist during the standard macroscopic pathological examination. Because the lesion was excised en bloc, the recorded specimen diameter included the cartilaginous cap together with a thin cortical margin intentionally removed during surgery.

**Figure 5:**
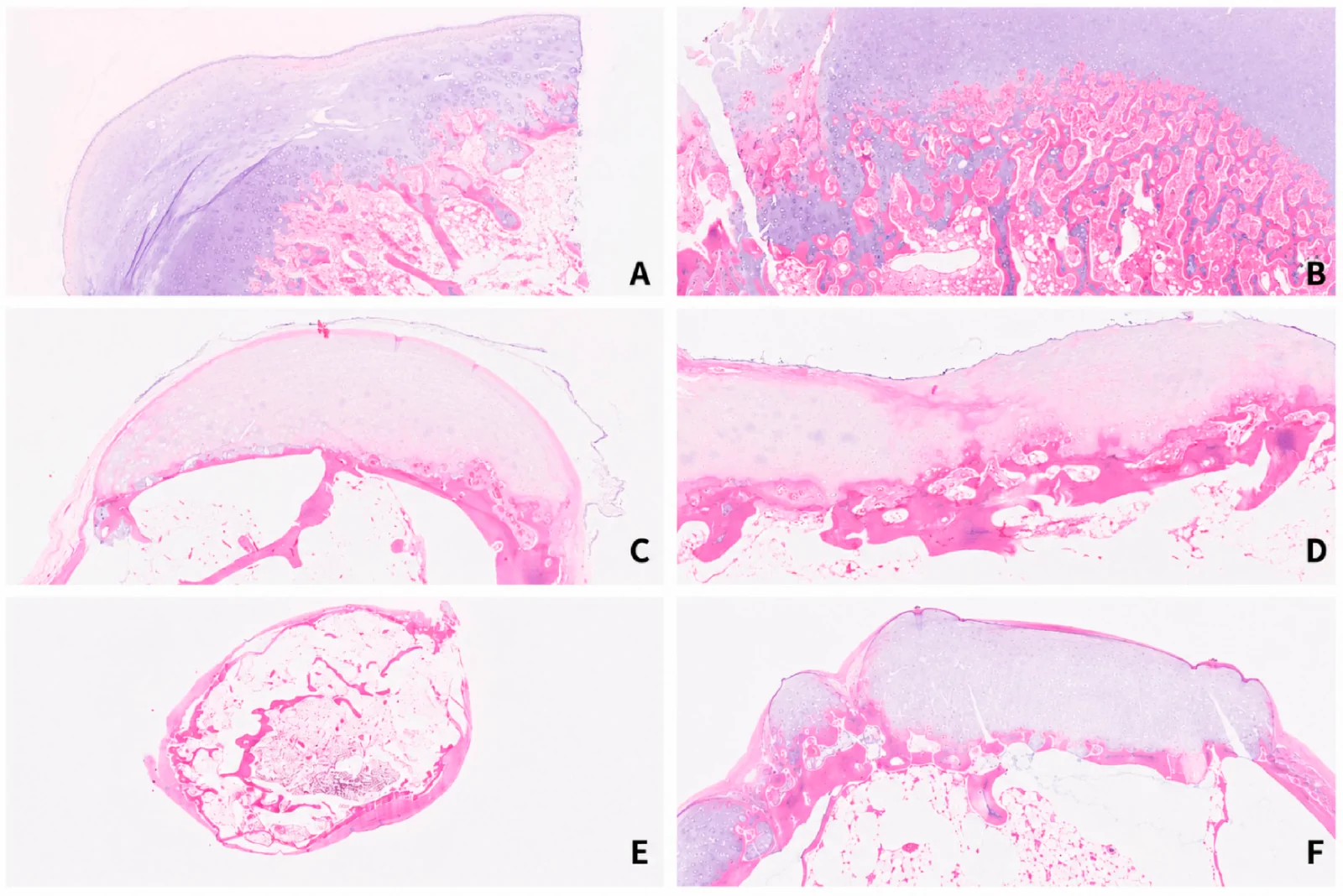
Histopathological features of osteochondroma: Representative hematoxylin and eosin (H&E)-stained sections demonstrating characteristic histopathological features of osteochondroma. (**A**,**C**) Low-power views (40× magnification) showing a cartilage cap overlying mature trabecular bone with continuity between the lesion and underlying bone. (**B**,**D**,**F**) High-power views (200× magnification) demonstrating endochondral ossification beneath the cartilage cap, with trabecular bone formation and marrow spaces. (**E**) Low-power view (20× magnification) showing the overall architecture of the lesion with cortical and medullary continuity.

Histologically, osteochondroma has a characteristic appearance. The lesion is composed of a cartilage cap of variable thickness resembling normal growth plate cartilage, with chondrocytes arranged in clusters within lacunae. Beneath the cartilage cap, endochondral ossification is present, with formation of mature trabecular bone. The bony trabeculae are lined by osteoblasts and contain intervening marrow spaces with fatty marrow and/or hematopoietic elements. In the present cohort, histopathological examination confirmed osteochondroma in all surgically treated patients (Figure 5).

### 2.7. Follow-Up

Follow-up was performed at the outpatient clinic 7–10 days after surgery for wound inspection and suture removal. Subsequent follow-up visits were scheduled at 1, 3, 6, and 12 months, and annually thereafter. At each visit, patients underwent clinical assessment focused on symptom resolution, wound healing, and detection of postoperative complications or recurrence. Routine postoperative radiographic imaging was not performed in asymptomatic patients. Imaging studies were obtained only when recurrence or other postoperative complications were clinically suspected.

### 2.8. Statistical Analysis

Statistical analysis was performed using appropriate descriptive and inferential methods. The normality of distribution of continuous variables was assessed using the Shapiro–Wilk test. Normally distributed variables are presented as mean ± standard deviation (SD), while non-normally distributed variables are presented as median and interquartile range (IQR). Categorical variables are expressed as absolute and relative frequencies (n and %). Comparisons between two independent groups were performed using the Mann–Whitney U test, while paired comparisons were assessed using the Wilcoxon signed-rank test. Associations between categorical variables were evaluated using the chi-square test. Correlations between continuous variables were assessed using Spearman’s rank correlation coefficient. Logistic regression analysis was performed to identify predictors of symptom occurrence, and results are presented as odds ratios (OR) with corresponding 95% confidence intervals (CI). A *p*-value < 0.05 was considered statistically significant. Statistical analyses were performed using Python (version 3.12.7, Python Software Foundation, Wilmington, DE, USA) with the SciPy (version 1.14.1), pandas (version 2.2.3), and statsmodels (version 0.14.4) libraries. The manuscript was prepared in accordance with the Strengthening the Reporting of Observational Studies in Epidemiology (STROBE) reporting guideline [15].

## 3. Results

A total of 75 patients with 76 exostoses were included in the analysis. All lesions were solitary osteochondromas. One patient presented with two separate lesions, resulting in a total of 76 exostoses. The median age was 13 years, with a predominance of males (64.0%). Most patients had no significant comorbidities and were classified as ASA I, indicating an overall healthy study population. Detailed demographic characteristics are presented in Table 1.

The majority of exostoses were located distally (73.7%), and pain was the most common symptom (63.2%). Symptom duration varied, with a median of 5 months. Radiographic measurements were consistently smaller than intraoperative specimen measurements (median 3.0 vs. 4.0 cm, *p* < 0.001). Detailed clinical and radiological characteristics of exostoses are presented in Table 2.

Since some patients presented with multiple symptoms and/or underwent more than one imaging modality, these variables were analyzed as non-mutually exclusive; therefore, their total exceeds the number of exostoses (*n* = 76). IQR—interquartile range.

**Figure 6:**
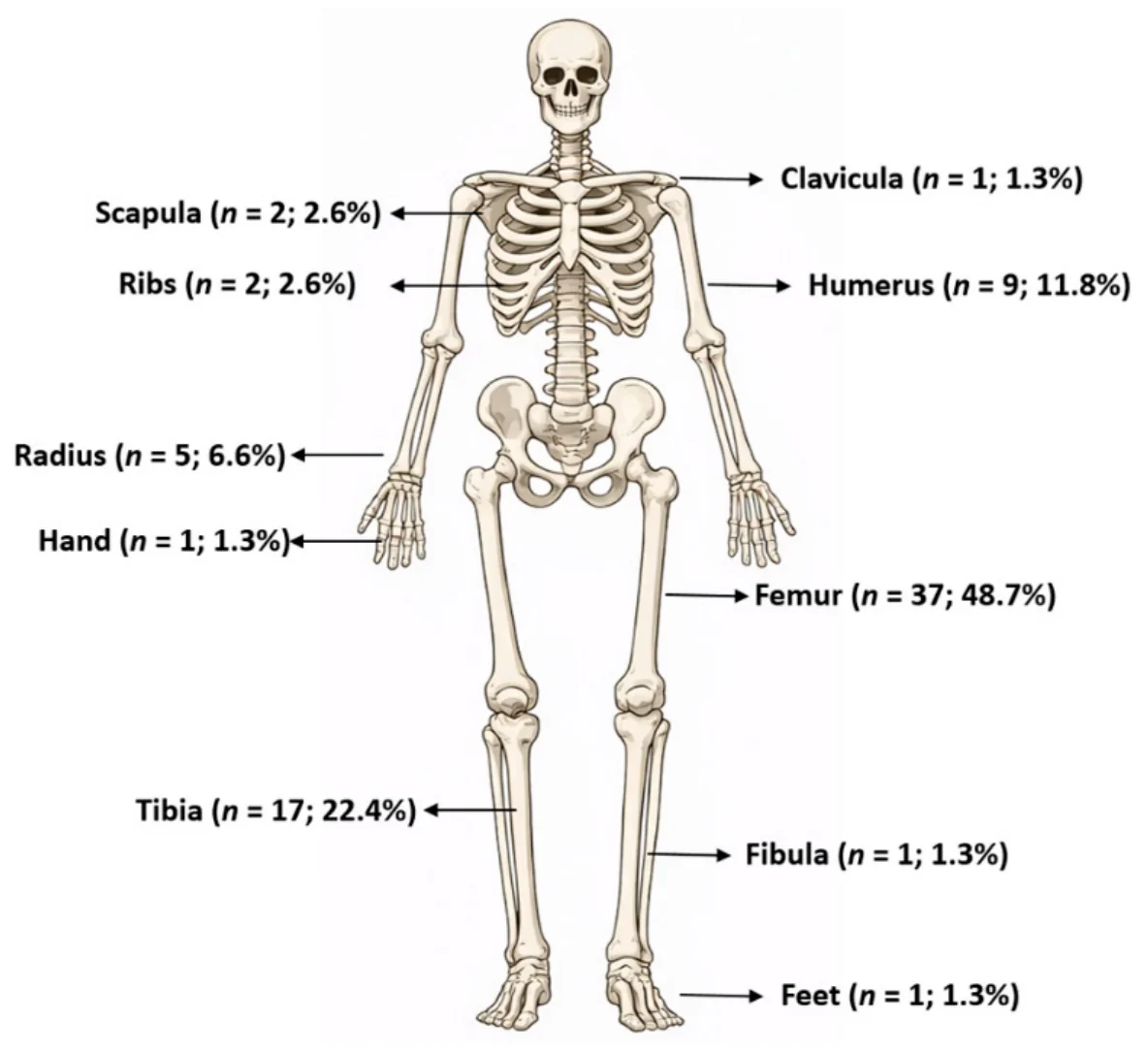
Distribution of exostoses by anatomical location (*n* = 76).

The distribution of exostoses according to the anatomical location is presented in Figure 6. The most common locations were the femur (48.7%) and tibia (22.4%), while other sites were considerably less frequent. Osteochondromas of the humerus accounted for 11.8% of cases, and those of the fibula for 6.6%. All other locations, including the scapula, ribs, hand, foot, clavicle, and ulna, were observed only sporadically or in a small number of cases.

Treatment outcomes are presented in Table 3. Most patients had an uneventful postoperative course (94.6%), with wound infection and hematoma occurring in 2.7% of cases each. According to the Clavien–Dindo classification, both cases of postoperative hematoma were classified as grade I, whereas both wound infections were classified as grade II. The median length of hospital stay was 2 days (IQR 2–2.5). No recurrences or reoperations were recorded during follow-up. The median follow-up duration was 63 months (IQR 27.5–91.0), and osteochondroma was histopathologically confirmed in all cases.

Additional analyses were performed to assess the association between clinical and morphological characteristics of exostoses (Table 4). No statistically significant difference in lesion diameter was observed between symptomatic and asymptomatic exostoses (*p* = 0.838). Similarly, no significant association was found between lesion location and the presence of pain (*p* = 0.250).

**Figure 7:**
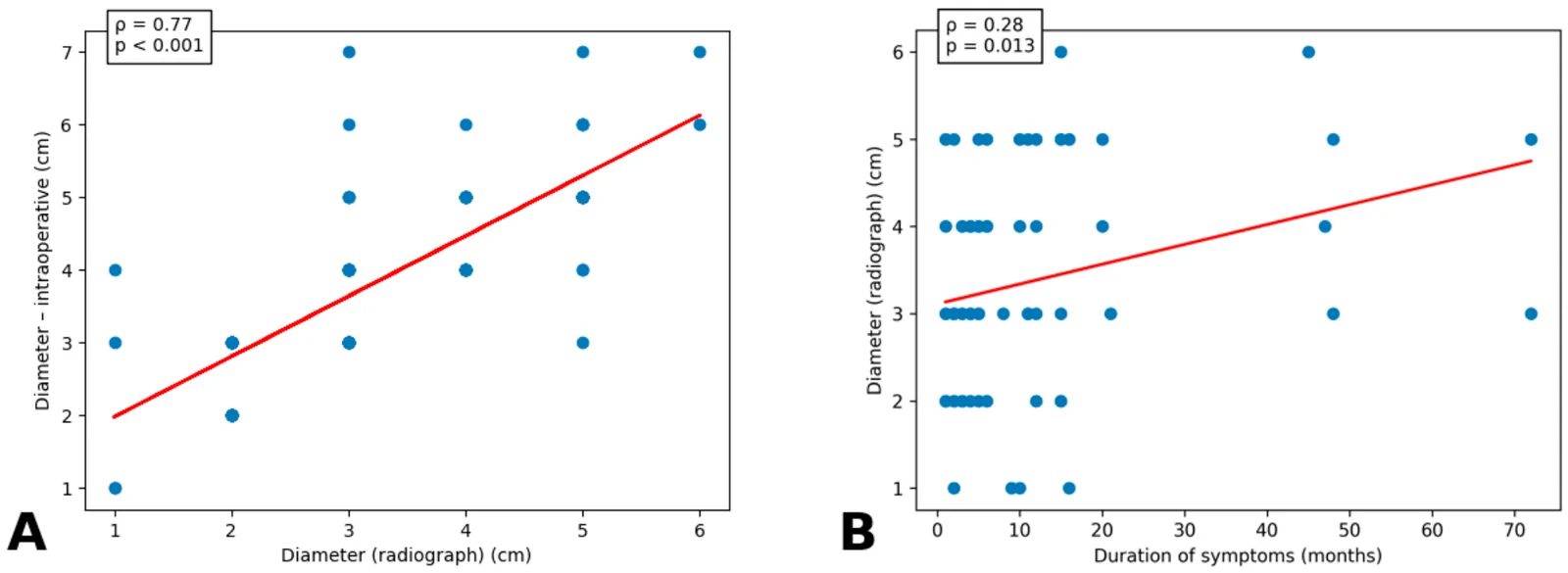
Relationships between clinical and morphological characteristics of exostoses: (**A**) Correlation between radiographic and intraoperative exostosis diameter; (**B**) Correlation between symptom duration and radiographic exostosis diameter.

A strong positive correlation was observed between radiographic and specimen measurements of exostosis diameter (ρ = 0.77; *p* < 0.001), indicating good agreement between the two measurement methods. Intraoperative specimen measurements were consistently larger than radiographic measurements. Furthermore, a weak but statistically significant positive correlation was observed between symptom duration and exostosis diameter (ρ = 0.28; *p* = 0.013), indicating that patients with longer symptom duration tended to present with larger lesions at the time of surgery (Figure 7). In addition, the Wilcoxon signed-rank test confirmed that specimen diameter was significantly greater than radiographic diameter (median 4.0 cm vs. 3.0 cm; *p* < 0.001).

In this exploratory logistic regression analysis, none of the evaluated variables (exostosis diameter, patient age, lesion location, and sex) were significantly associated with symptomatic presentation. However, these findings should be interpreted cautiously because the study cohort consisted exclusively of surgically treated patients, resulting in a selected population with a high prevalence of symptomatic lesions and limited variability in the outcome of interest. Consequently, the analysis should be regarded as hypothesis-generating rather than definitive, and larger studies including both surgically and conservatively managed patients are needed to identify reliable predictors of symptomatic presentation (Table 5).

## 4. Discussion

The primary aim of our study was to analyze the clinical characteristics and outcomes of surgical treatment of osteochondromas in children and adolescents during the study period at a tertiary pediatric center. Our findings indicate that patients were most commonly adolescents, with a predominance of males, and that osteochondromas were most frequently located in the distal femur and proximal tibia. Pain was the most common presenting symptom and the leading indication for surgical intervention. Surgical excision of symptomatic osteochondromas proved to be a safe treatment modality, with a low complication rate and no recurrences or need for reoperation during a relatively long follow-up period. Standard radiography showed good correlation with intraoperative measurements of lesion size, although it consistently underestimated the true size of osteochondromas. Furthermore, lesion size, location, patient age, and sex were not identified as significant predictors of symptomatic presentation, suggesting a multifactorial basis for symptom development.

Our demographic and anatomical findings are consistent with previously published studies describing osteochondroma as the most common benign bone tumor in the pediatric and adolescent population. In our cohort, patients were predominantly adolescents, with a male predominance, in line with recent pediatric series reporting that osteochondromas are most frequently diagnosed between 10 and 14 years of age and occur more commonly in males. In one such study, the median age at diagnosis was 11.4 years, males accounted for 62.7% of patients, and the distal femur and proximal tibia each represented 26.9% of all osteochondroma locations. Similar age distribution and lesion localization have been reported previously [2,16].

In our study, pain was the most frequent symptom and the most common indication for surgical treatment. Cosmetic concerns, mechanical symptoms, limitation of range of motion, and neurovascular compression were considerably less frequent but clinically relevant indications for surgery. These findings highlight the heterogeneity of clinical presentation and reinforce that the decision to operate should be individualized and based on the overall clinical context rather than solely on morphological characteristics of the lesion. Similar variability in presentation has been described by other authors, noting that symptoms depend largely on the anatomical location and relationship of the lesion to surrounding structures [17]. This is further illustrated by reports of osteochondromas in rare locations, such as the acromioclavicular joint and the talus, where symptoms result from compression and irritation of adjacent structures [18,19].

An exploratory analysis did not identify a statistically significant association between osteochondroma size and symptomatic presentation, and logistic regression did not demonstrate significant effects of age, sex, lesion location, or lesion diameter. However, these findings should be interpreted with caution because the present study included only surgically treated patients, representing a selected cohort with a predominance of symptomatic lesions. Consequently, the limited variability in the outcome reduces the ability of the model to detect meaningful predictors and precludes definitive conclusions regarding factors associated with symptom development. Nevertheless, our findings suggest that lesion diameter alone is unlikely to fully explain clinical presentation. Rather, symptom occurrence probably depends on multiple factors, including the anatomical relationship of the lesion to adjacent tendons, muscles, nerves, and blood vessels, as well as the biomechanical demands of the affected region. This interpretation is supported by previous reports describing deformities, fractures, vascular and neurological complications, and bursitis as consequences of local anatomical relationships rather than lesion size alone [9,20,21]. Likewise, osteochondromas arising in atypical locations frequently become symptomatic because of mechanical interaction with surrounding structures regardless of their absolute dimensions [10,22,23]. Although our findings are consistent with this concept, they should be regarded as exploratory and require confirmation in larger studies including both surgically and conservatively managed patients. Similar conclusions have been reported by authors who emphasize the benefits of surgical treatment in symptomatic patients and the option of conservative management in asymptomatic lesions, particularly in skeletally immature patients where spontaneous regression has also been described [7,24].

One of the more notable findings of our study was the discrepancy between radiographically estimated and intraoperatively measured osteochondroma size. Standard radiography was the primary diagnostic modality in nearly all patients and demonstrated good correlation with intraoperative measurements. Nevertheless, radiographic measurements were consistently smaller than intraoperative specimen measurements. This finding should be interpreted with caution because the two methods do not assess identical anatomical constructs. Plain radiographs primarily depict the ossified component of the lesion, whereas the surgically excised specimen additionally includes the cartilaginous cap and a thin cortical margin intentionally removed during surgery to minimize the risk of recurrence. Consequently, the observed difference cannot be attributed solely to limitations of plain radiography. In addition, uncorrected radiographic magnification may have contributed to the measurement discrepancy. Unfortunately, cartilage cap thickness was not routinely recorded on histopathological examination in our cohort; therefore, we were unable to determine the relative contribution of the cartilage cap to the observed difference. Nevertheless, our findings highlight that measurements obtained from plain radiographs and surgical specimens should not be interpreted as directly interchangeable. Previous studies have similarly demonstrated that CT and particularly MRI provide more accurate assessment of the cartilage cap and overall lesion morphology, with MRI being especially valuable when detailed evaluation of lesion extent, cartilage cap thickness, or the relationship to surrounding structures is required [25]. Surgical treatment in our cohort yielded very favorable outcomes. The majority of patients experienced no postoperative complications, while surgical site infection and hematoma were observed in a small number of cases. During a median follow-up of 63 months, no recurrences or need for reoperation were recorded, indicating high effectiveness of surgical excision in the treatment of symptomatic osteochondromas in children and adolescents. These results are consistent with previous studies reporting low complication rates and excellent functional outcomes following surgical treatment. Wu et al. reported only 2% major complications after surgery for periarticular knee osteochondromas in pediatric patients [26], while Bottner et al. observed symptom resolution in 93.4% of patients with low postoperative morbidity [27]. Additional support comes from a multicenter pediatric study of spinal osteochondromas, in which no recurrences or disease-related complications were observed during follow-up [13]. The absence of recurrence in our cohort is likely attributable to complete surgical excision, including removal of the cartilage cap and perichondrium. Since recurrence is most commonly associated with incomplete excision, our findings further support the safety of surgery when performed with appropriate indication and technique.

The results of our study have important clinical implications, providing additional data on surgically treated osteochondromas in the pediatric and adolescent population with relatively long follow-up. The median follow-up of 63 months allowed reliable assessment of postoperative outcomes and further confirmed the safety and efficacy of surgical treatment. Our findings suggest that the decision to operate should not be based solely on lesion size. Given the lack of significant association between lesion diameter and symptom presence, greater importance appears to lie in lesion location, its relationship to surrounding anatomical structures, and its functional and cosmetic impact on the patient. An individualized approach is therefore essential in clinical decision-making. From a diagnostic perspective, plain radiography remains the primary imaging modality due to its availability and ability to demonstrate characteristic features. However, in cases of larger lesions, atypical locations, symptom progression, or suspected complications, additional imaging, particularly MRI, should be considered, as it allows more detailed evaluation of the cartilage cap and surrounding structures. This is especially important in lesions of the axial skeleton and spine, where MRI and CT play a key role in assessing relationships with neural structures, differentiating tumor-related from degenerative causes of symptoms, and planning surgical treatment [28,29,30].

It is important to note that the true prevalence of osteochondromas is likely underestimated, as many lesions remain asymptomatic and undiagnosed. In one osteological study, the prevalence was reported as 0.44%, which is lower than earlier estimates and suggests possible selection bias in clinical and radiological series toward symptomatic patients [31]. Furthermore, recent studies indicate that osteochondromas—particularly in patients with hereditary multiple exostoses—may significantly impact quality of life, daily functioning, and chronic pain [3]. Despite advances in understanding the molecular basis of the disease, the relationship between genetic features, clinical presentation, and symptom severity remains incompletely understood [6].

This study has several limitations that should be considered when interpreting the results. First, it is a retrospective single-center study, and the findings may not fully reflect the experience of other institutions or patient populations. Second, only surgically treated patients were included, limiting the generalizability of the results to the broader population of children with osteochondromas. A substantial proportion of lesions remain asymptomatic and are either incidentally detected or managed conservatively, meaning that surgical series do not capture the full disease spectrum. Interobserver and intraobserver reliability of radiographic measurements was not formally assessed because measurements were performed retrospectively as part of the study protocol. Additionally, the relatively small number of patients with less common anatomical locations limited more detailed region-specific analyses. Finally, functional outcomes and quality of life were not assessed using standardized, validated functional or patient-reported outcome measures. Consequently, the true clinical benefit of surgery beyond the absence of complications and recurrence could not be comprehensively evaluated. Future studies should incorporate validated functional assessment instruments to provide a more comprehensive evaluation of treatment success.

Future research should focus on prospective, multicenter studies with larger cohorts, including both surgically and conservatively managed osteochondromas. Standardization of surgical indications, as well as methods for assessing cartilage cap thickness, functional outcomes, pain, cosmetic satisfaction, and quality of life, would be particularly valuable. Further investigation is also needed to identify factors that best predict symptom development and the need for surgical intervention, as well as to develop more precise risk stratification and decision-making models. Despite these limitations, our findings demonstrate that surgical excision of symptomatic osteochondromas in children and adolescents is a safe procedure with low rates of complications and recurrence. However, given the study design and patient selection, the results should be interpreted with caution and cannot be fully generalized to the entire pediatric population with osteochondromas.

## 5. Conclusions

Surgical excision of symptomatic osteochondromas in children and adolescents represents a safe treatment strategy, associated with excellent outcomes, low complication rates, and no observed recurrence in our cohort. In this surgically treated cohort, no significant associations were identified between lesion size, age, sex, or lesion location and symptomatic presentation. Because of the selected nature of the study population, these findings should be considered exploratory and require confirmation in larger studies including both surgically and conservatively managed patients. Standard radiography correlated well with intraoperative specimen measurements but yielded systematically smaller values, reflecting differences between radiographic visualization and the surgically excised specimen rather than solely an imaging limitation. Postoperative follow-up was primarily based on clinical assessment, and routine imaging was not performed in asymptomatic patients. Therefore, asymptomatic recurrences may have remained undetected, and the true recurrence rate could have been slightly underestimated. Overall, treatment decisions should be guided primarily by symptoms and functional impairment rather than radiological dimensions alone.

## Figures and Tables

**Table 1:** Demographic characteristics of patients (*n* = 75).

Variable	Value
Age, years; median (IQR)	13.0 (11.0–15.0)
Sex; n (%) Male Female	48 (64.0%) 27 (36.0%)
Height, cm; median (IQR)	165.0 (152.0–174.0)
Weight, kg; mean (SD)	51.16 (15.72)
BMI, kg/m^2^; median (IQR)	19.1 (17.1–20.6)
Comorbidities; n (%)	3 (4%)
ASA classification; n (%) ASA I ASA II	72 (96%) 3 (4%)

BMI—Body Mass Index; ASA—American Society of Anesthesiologists; IQR—Interquartile range; SD—Standard deviation.

**Table 2:** Clinical and radiological characteristics of exostoses (*n* = 76).

Variable	Value
Localization; n (%) Proximal Distal	20 (26.3%) 56 (73.7%)
Symptoms; n (%) Pain Mechanical symptoms Cosmetic concern Neurovascular compression Restricted range of motion	48 (63.2%) 9 (11.8%) 25 (32.9%) 5 (6.6%) 8 (10.5%)
Symptom duration, months; median (IQR)	5.0 (3.0–12.0)
Exostosis diameter—imaging, cm; median (IQR)	3.0 (2.5–4.0)
Exostosis diameter—intraoperative, cm; median (IQR)	4.0 (3.0–5.0)
Imaging modalities; n (%) Standard radiography Computed tomography Magnetic resonance imaging	75 (98.7%) 4 (5.3%) 6 (7.9%)

**Table 3:** Treatment outcomes of patients with osteochondroma (*n* = 75).

Variable	Value
Complications; n (%) No complications Wound infection Hematoma	71 (94.6%) 2 (2.7%) 2 (2.7%)
Length of hospital stay, days; median (IQR)	2.0 (2.0–2.5)
Recurrence; n (%)	0 (0.0%)
Reoperation; n (%)	0 (0.0%)
Follow-up, months; median (IQR)	63.0 (27.5–91.0)
Histopathological diagnosis—osteochondroma; n (%)	75 (100.0%)

IQR—interquartile range.

**Table 4:** Association between clinical, morphological, and radiological variables.

Analysis	Test	*p*
Exostosis diameter vs. presence of symptoms	U = 653	0.838
Exostosis location vs. presence of symptoms	χ^2^ = 1.33	0.250
Radiographic vs. intraoperative exostosis diameter	ρ = 0.77	<0.001
Symptom duration vs. exostosis diameter	ρ = 0.28	0.013

**Table 5:** Logistic regression analysis of predictors of symptom occurrence.

Variable	OR	95% CI	*p*
Exostosis diameter (cm)	0.84	0.55–1.26	0.394
Age (years)	1.12	0.97–1.30	0.121
Distal location	2.32	0.75–7.16	0.145
Male sex	0.64	0.22–1.86	0.411

OR—odds ratio; CI—confidence interval.

## Data Availability

The original contributions presented in this study are included in the article. Further inquiries can be directed to the corresponding author.
